# Cavitary Lung Abscess Secondary to a Tracheal Bronchus: A Pediatric Patient With Noonan Syndrome

**DOI:** 10.7759/cureus.91179

**Published:** 2025-08-28

**Authors:** Andrea M Lauffer, Sundus Ghouri, Jacob T Kilgore, Jeff Harris, Genevra Addis

**Affiliations:** 1 Department of Pediatric Hospital Medicine, Marshall University Joan C. Edwards School of Medicine, Huntington, USA; 2 Department of Pediatrics, Marshall University Joan C. Edwards School of Medicine, Huntington, USA; 3 Department of Pediatric Infectious Diseases, Marshall University Joan C. Edwards School of Medicine, Huntington, USA; 4 Department of Pediatric Cardiology, Marshall University Joan C. Edwards School of Medicine, Huntington, USA

**Keywords:** diagnostic ct imaging, lung cavitation, noonan syndrome, pediatric clinical cardiology, pediatric hospital medicine, pediatric infectious disease, tracheal bronchus

## Abstract

A 13-year-old male with a past medical history of Noonan syndrome, pulmonary valve stenosis status post balloon valvuloplasty and subsequent surgical valvotomy with main pulmonary artery augmentation, splenomegaly, and von Willebrand disease type 1 presents with chest pain, fever, and chronic, recurrent vomiting. At the outlying facility, computed tomography of the chest revealed a right upper lobe pulmonary cavitation. After an extensive workup, he was found to have a right-sided tracheal bronchus via bronchoscopy. Broad-range polymerase chain reaction analysis of the biopsied specimen demonstrated polymicrobial organisms consistent with an organized pneumonia identified upon pathological review. He was treated with six weeks of antibiotic therapy and made a full recovery. The development of his pulmonary cavitation was attributed to his anatomic abnormality of the tracheal-bronchial tree as well as chronic vomiting with aspiration, thought to be secondary to his significant splenomegaly (18.8 x 13.8 x 6.4 cm). The presence of a tracheal bronchus is rare in the pediatric population, and patients often remain asymptomatic. However, co-existing tracheobronchial anomalies are often found in patients with underlying congenital heart disease (CHD). Therefore, in patients with CHD and new-onset atypical pulmonary pathology, a multidisciplinary approach should commence to evaluate for possible tracheobronchial abnormalities. If identified, prompt recognition, treatment, and prevention measures should be used to prevent future pathology.

## Introduction

A 13-year-old male with a past medical history of Noonan syndrome, pulmonary valve stenosis status post balloon valvuloplasty and subsequent pulmonary valvotomy with main pulmonary artery augmentation, splenomegaly, and von Willebrand type I presented as a transfer from an outlying facility for fever and productive cough. He also had a history of chronic vomiting. After an extensive clinical evaluation utilizing diagnostic laboratory, radiologic, and surgical methods, the patient was diagnosed with a polymicrobial pulmonary cavitary lesion with organized pneumonia. It was discovered that the patient had an undiagnosed tracheal bronchus. This presented as a major risk factor for the patient to develop a severe pulmonary infection. The majority of tracheal bronchus cases are diagnosed incidentally by bronchoscopy with an estimated incidence of 0.9%-3% in pediatric patients [1]. A tracheal bronchus diagnosis has been associated with other congenital abnormalities such as Down syndrome/trisomy 21, VATER/VACTERL syndrome, tracheoesophageal (TE) fistula, esophageal atresia, laryngeal and duodenal webs, spinal fusion defects, hypoplastic lung, and congenital heart disease (CHD) [1]. However, there is a paucity of data establishing an association between Noonan syndrome and tracheal bronchus despite Noonan syndrome's association with CHD, notably pulmonary pathology. 

Verbal informed consent via telephone for publication of clinical details and images was obtained from the patient's legal guardian. 

## Case presentation

**Figure 1:**
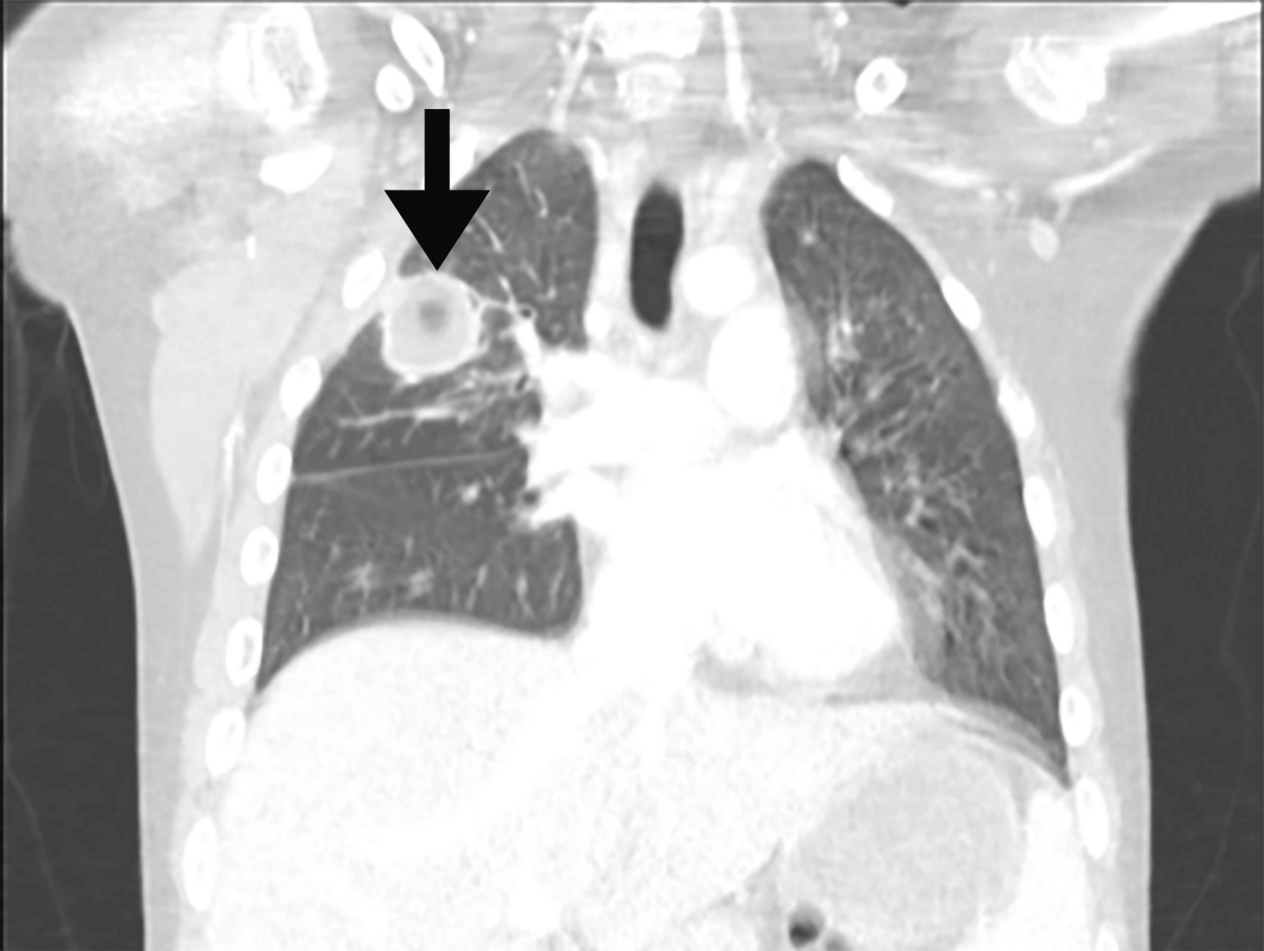
Computed tomography image of right upper lobe cavitary lesion

Prior to admission, the patient had been complaining of right-sided chest and subscapular pain as well as chronic, recurrent, post-prandial emesis for which a gastroenterology referral was pending. Upon presentation, the patient was febrile (Tmax 102.7°F) but otherwise hemodynamically stable on room air. Laboratory data revealed an elevated white blood cell count and C-reactive protein (Table 1). The respiratory pathogen polymerase chain reaction panel was positive for rhinovirus/enterovirus. An electrocardiogram was unrevealing. Chest X-ray showed a 3.6 cm round opacity in the right upper lobe with central lucency. Follow-up computed tomography (CT) chest with intravenous contrast demonstrated a rounded, cavitary mass-like consolidation measuring approximately 3.0 x 3.4 cm in the right upper lung field (Figure 1). Just inferior along the right major fissure was an additional consolidation containing a small cavitation. Additional scattered tree-in-bud opacities were seen in the posterior right upper field. Extensive bilateral supraclavicular, mediastinal, and hilar lymphadenopathy was also noted.

**Table 1:** Initial admission laboratory data

Laboratory marker	Units	Patient values	Reference range
White blood cell count	cells/microliter	13,500	4800-10,800 cells/microliter
C-reactive protein	mg/dL	3.3	<0.3 mg/dL
Alanine transaminase	U/L	7	7-56 U/L
Aspartate aminotransferase	U/L	11	8-50 U/L
Procalcitonin	ng/mL	0.07	<0.05 ng/mL
Troponin	ng/mL	< 2.7	< 2.7 ng/mL

**Figure 2:**
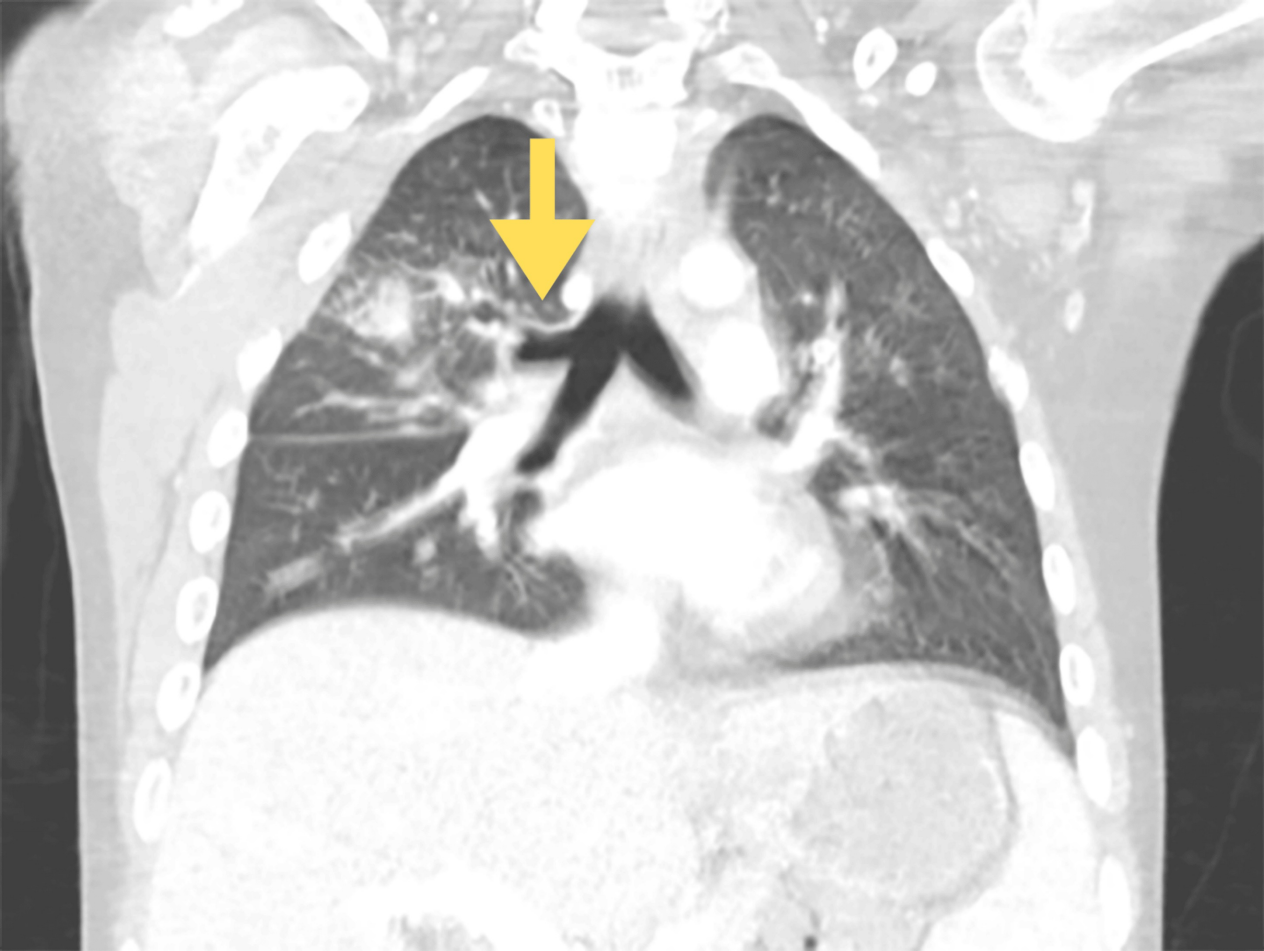
Computed tomography image of right-sided tracheal bronchus

The patient was placed in airborne precautions for possible mycobacterial disease, started on empiric piperacillin-tazobactam, and an extensive infectious diseases workup commenced under the direction of the pediatric infectious diseases (Peds ID) team. Initial testing was negative for bacteremia, pulmonary *Mycobacterium tuberculosis* (three consecutively negative sputum acid-fast bacillus stains/cultures), and endemic fungi such as histoplasmosis and blastomycosis. Transthoracic echocardiogram showed mild pulmonary valve regurgitation, unchanged from baseline. The cardiothoracic surgery team was consulted and completed a bronchoscopy with bronchoalveolar lavage (BAL). BAL fluid was non-diagnostic, including negative bacterial, fungal, and mycobacterial cultures, and cytology was negative for malignancy. However, bronchoscopy was notable for the upper lobe bronchus take-off from the main carina, consistent with a right-sided tracheal bronchus (Figure 2). 

For further diagnostic and therapeutic evaluation, the Peds ID team recommended a biopsy of the cavitary lesion with planned broad-range polymerase chain reaction infectious testing. Patient underwent CT-guided core biopsy of the right upper lobe cavitary lesion. Results of the biopsy showed no malignant appearance, but with marked acute and chronic inflammation and focal organization, consistent with pneumonia. Broad range polymerase chain reaction testing demonstrated evidence of polymicrobial bacterial involvement, including *Streptococcus intermedius*, *Cutibacterium acnes*, and *Corynebacterium parakroppenstedtii*. Antibiotic therapy was narrowed to amoxicillin-clavulanic acid, and the patient's two-week hospital course was uncomplicated. The patient completed an extended (~ four weeks) amoxicillin-clavulanic acid course with normalization of inflammatory markers and imaging through close outpatient evaluation. An elective splenectomy is planned due to worsening splenomegaly in association with chronic, recurrent post-prandial emesis and an ongoing aspiration risk from the tracheal bronchus. Referrals to pediatric pulmonology and gastroenterology, as well as a re-referral to pediatric cardiology, were also recommended.

## Discussion

A tracheal bronchus most commonly arises on the right side and can typically be found within 2 cm of the carina; however, evidence suggests a tracheal bronchus can arise anywhere from the cricoid cartilage to the carina [1]. It has been reported that ~90% of cases involving a tracheal bronchus are associated with other pathologies, with ~70% of those cases being cardiac in origin, including Tetralogy of Fallot, ventricular septal defect, aortic arch abnormalities, great vessel transposition, and pulmonary vascular malformations [2,3]. 

Multi-detector CT (MDCT) with 3D image reconstruction is the gold-standard test in detecting congenital tracheobronchial anomalies as it allows the making of a direct diagnosis of tracheal bronchus non-invasively [1]. One study demonstrated that CT scans prior to bronchoscopies detected only 53.3% of tracheal bronchi, highlighting the importance of image analysis to diagnose the presence of the tracheal bronchus [3]. A delay in diagnosis rate has been reported at 4.1% [4]. Another study showed that chest CT was able to detect tracheal bronchus anomaly in six out of eight cases, supporting the recommendation that 3D imaging with CT scanning should be the first-line method of diagnosis [5]. Our patient's initial CT analysis did not diagnose a tracheal bronchus, but it was found upon retrospective provider review following gross identification during bronchoscopy.

## Conclusions

Based on this novel case, in patients with CHD and new onset, atypical pulmonary pathology, a multidisciplinary approach should commence to evaluate for potential contributory tracheobronchial abnormalities. Diagnostic approach should be tiered towards utilizing CT imaging as the gold standard for primary diagnosis. Once identified, prompt recognition, treatment, and prevention measures should be used to prevent future pulmonary pathology.
